# Modulation of Antimicrobial Resistance in *Listeria monocytogenes* via Synergistic Interactions Between *Thymbra capitata* L. (Cav.) Essential Oil and Conventional Antibiotics

**DOI:** 10.3390/antibiotics14060623

**Published:** 2025-06-19

**Authors:** Francesca Maggio, Francesco Buccioni, Stefania Garzoli, Antonello Paparella, Annalisa Serio

**Affiliations:** 1 Department of Bioscience and Technology for Food, Agriculture and Environment, University of Teramo, 64100 Teramo, Italy; fmaggio@unite.it (F.M.); fbuccioni@unite.it (F.B.); apaparella@unite.it (A.P.); 2 Department of Drug Chemistry and Technology, Sapienza University, 00185 Rome, Italy; stefania.garzoli@uniroma1.it

**Keywords:** *Listeria monocytogenes*, essential oil, antimicrobial resistance, antibiotics, interactions

## Abstract

Background/Objectives: Antimicrobial resistance (AMR) poses a significant global health challenge, contributing to foodborne infections and diminishing the effectiveness of conventional antibiotics. In the quest for alternative strategies to mitigate resistance, this study has assessed the potential of *T. capitata* L. (Cav.) essential oil (TEO) to boost the antibiotic efficacy on *L. monocytogenes*. Methods: Five *L. monocytogenes* strains of different origins were tested with TEO alone and in combination with gentamicin, ampicillin, and penicillin G. Moreover, the cells were exposed to sublethal concentrations of TEO for 1 h to evaluate the effects on the antibiotic effectiveness. The antimicrobial activity was assessed by determining the Minimum Inhibitory (MICs) and Bactericidal Concentrations (MBCs), while potential interactions were evaluated using the Fractional Inhibitory Concentration Index and by studying the cell growth dynamics. Results: TEO demonstrated inhibitory activity against *L. monocytogenes* strains, both alone, in pre-exposure, and in combination with antibiotics, causing up to a seven-fold reduction in MIC and MBC values (from 8 to 1 µg/mL) and restoring susceptibility to the antimicrobial treatments. Positive interactions between TEO and antibiotics were observed, particularly for clinical isolates. Conclusions: TEO could be a promising antibiotic adjuvant in antimicrobial treatments, offering a natural and effective strategy to enhance antibiotic efficacy and to counteract resistance in *L. monocytogenes*.

## 1. Introduction

Antimicrobial resistance (AMR) is a complex and multifaceted phenomenon that arises from the evolutionary processes and genetic adaptations of bacteria, fungi, parasites, and viruses [1]. Through such adaptations, microorganisms have different ways to reduce the effectiveness of antimicrobial agents such as antibiotics, antifungals, antivirals, and antiparasitics that have conventionally been used in prophylaxis and the treatment of infectious diseases. The overuse and misuse of antibiotics in the human and animal sectors, as well as in the agricultural chain, significantly increases the dissemination of bacterial resistance genes, causing a “Silent Pandemic” that has the potential to result in approximately 10 million fatalities by the year 2050 [2]. Internal bacterial mechanisms contribute to antibiotic resistance; nonetheless, a complex network of several factors influences the development of resistance. Bacteria acquire antimicrobial resistance through different mechanisms: mutations at the target site, the enzymatic modification or degradation of antibiotics, active efflux pumps, reduced permeability to antibiotics that limit access to target sites, the acquisition of alternative metabolic pathways, and modifications of metabolic activities [3].

Along with many other pathogenic bacteria, *Listeria monocytogenes* can be resistant to a wide range of antimicrobial compounds due to the acquisition of resistance genes via horizontal gene transfer, mainly from other species within its genus and from other genera such as *Enterococcus*, *Staphylococcus*, and *Bacillus* spp. [4]. Resistance in *L. monocytogenes* primarily involves inert elements such as gene-associated or intrinsic mechanisms that reduce the effectiveness of compounds, including efflux pumps, drug inactivation through restricted uptake, direct modifications of drugs or antibiotics, and the cellular target sites’ alteration to overcome drug entry [4], or metabolic mechanisms influenced through the regulation of enriched genes associated with biological functions such as transmembrane transport [5]. The rise in AMR in *L. monocytogenes* is a worldwide concern due to its association with severe foodborne infections, which contribute to significant global outbreaks of *L. monocytogenes* caused by contaminated food [6]. A multifaceted approach is required for the management of listeriosis, whereby the reference treatment for listeriosis typically involves the use of β-lactams in combination with an aminoglycoside [4], while in patients with β-lactam allergies, cotrimoxazole (trimethoprim-sulfamethoxazole) is commonly employed [7]. Consequently, the misuse of the conventional antibiotics for treating life-threatening and invasive infections has led to an increased resistance in *L. monocytogenes* throughout time [7,8]. The primary cause of high-level trimethoprim resistance in *L. monocytogenes* is the replacement of a trimethoprim-sensitive dihydrofolate reductase by plasmids, transposons, or a cassette-borne version, also due to the presence of the *dfrD* resistance-encoding gene [7]. In contrast, penicillin G resistance in *L. monocytogenes* is linked to the *penA*-encoding gene; penA is a penicillin-binding protein (PBP) that functions as an enzyme that elongates glycan chains in peptidoglycan and facilitates peptide cross-linking between these chains. Multiple PBPs were identified in *Listeria* that confer a natural resistance phenotype [7]. Similarly, Tn3706 on plasmid pIP501, which is a composite transposon containing the gentamicin resistance gene *aac6′*-*aph2*, revealed in *L. monocytogenes* aminoglycoside resistance genes, encodes gentamicin-inactivating enzymes because of transposition with *Streptococcus*, *Enterococcus*, and *Staphylococcus* species [9].

Therefore, the management of listeriosis is a complex task that would benefit from complementary therapeutic approaches. Natural compounds, such as EOs, offer promising solutions due to their different mechanisms of action and lower likelihood of resistance development, as they target bacterial cells in a multidimensional manner. The primary mechanisms include the disruption of cell membranes via changes in fatty acid composition and the destruction of cellular structures [10]. Moreover, the reduction in proton motive force, the depletion of ATP, and the leakage of metabolites and ions collectively induce cellular death [10]. A potential mechanism of action may involve the modification of bacterial metabolism. For example, the exposure to sub-lethal concentrations of *Thymbra capitata* L. (Cav.) hydrolate resulted in a clear lag phase extension in *L. monocytogenes* cells in the presence of different carbohydrates, including glucose, lactose, trehalose, cellobiose, mannitol, and fructose [11], through the downregulation of the Lmo2373 and phosphocarrier HPr proteins, involved in carbohydrate transport and metabolism (unpublished data). Furthermore, natural compounds may boost the activity of conventional antibiotics by restoring susceptibility in bacteria, suggesting a potential approach to counteract AMR. Cells of *L. monocytogenes* exposed to 1.25 μL/mL of *Origanum vulgare* L. EO were inhibited in the presence of lincomycin and vancomycin, whereas untreated cells were able to grow [12]. Synergistic and additive interactions between EOs and antibiotics against foodborne pathogens have been documented across multiple scientific investigations, although the underlying mechanisms of action are not fully elucidated yet [13,14,15,16]. However, several studies have demonstrated synergistic effects of EO compounds combined with antibiotics against Gram-negative and -positive bacteria through significant reductions in inhibitory concentrations, whereby the synergistic mechanisms have been primarily attributed to carvacrol’s multifaceted antimicrobial action, including bacterial membrane disruption, efflux pump inhibition, ATP metabolism interference, and the mitigation of antibiotic-induced oxidative stress [14,17,18,19].

Therefore, the aim of this study was to investigate whether the exposure of *L. monocytogenes* cells to TEO could affect their antibiotic susceptibility to gentamicin, ampicillin, and penicillin G. Five different *L. monocytogenes* strains of different origins were examined. The potential synergistic interactions between the EO and the antibiotic compounds were evaluated by calculating the Fractional Inhibitory Concentration Index (FICI) after 48 h of incubation at 37 °C. The effect of the pre-exposure of the cells to sub-lethal concentrations of TEO on *L. monocytogenes* susceptibility to the antibiotics was assessed by determining Minimum Inhibitory Concentrations (MICs) and Minimum Bactericidal Concentrations (MBCs). Observed outcomes were organised in a graphic matrix to identify relevant patterns and interpret the inherent interaction between conventional antibiotics and TEO.

## 2. Results

### 2.1. Chemical Volatile Composition

The chemical composition of TEO was determined by GC-MS analysis, and 29 volatile components were identified (Table 1). The main detected compounds belonged to the terpene class, with monoterpenes prevailing over sesquiterpenes. Carvacrol was the most abundant component (55.4%), followed by *β*-caryophyllene (8.6%), *p*-cymene (7.7%), and *γ*-terpinene (7.6%). *β*-pinene, 1,8-cineole, *cis*-*β*-ocimene, *p*-cymenene, neral, aromadendrene, *δ*-cadinene, and isoaromadendrene epoxide were the least abundant compounds, exhibiting the same percentage mean values equal to 0.1%.

### 2.2. Antimicrobial Activity of T. capitata L. (Cav.) Essential Oil and Antibiotic Compounds Based on Minimum Inhibitory and Bactericidal Concentrations

Table 2 shows the MICs and MBCs of TEO and the antibiotics ampicillin, gentamicin, and penicillin G, determined against *L. monocytogenes* strains at 37 °C after 48 h of incubation (as reported in Section 4.3). Results obtained after 24 h were not included in tables, as they were identical to those observed at 48 h. As shown, *L. monocytogenes* ATCC 7644 was susceptible to all antibiotics, exhibiting MIC and MBC values of 0.5 µL/mL after 48 h of treatment at 37 °C. In most cases, the antibiotic compounds exhibited antimicrobial activity at MIC and MBC values of 2 µL/mL after 48 h at 37 °C. However, in some instances, higher MICs and MBCs were required. Specifically, after 48 h at 37 °C, the MICs for ampicillin against *L. monocytogenes* strains 17 and 229 were 8 µL/mL. Additionally, results for penicillin G against *L. monocytogenes* after 48 h of treatment gave MIC and MBC values of 64 µL/mL and 4 µL/mL for strains 6 and 229, respectively. Consequently, except for ATCC 7644, the *L. monocytogenes* strains analysed were classified as resistant to ampicillin, gentamicin, and penicillin G compounds tested, according to the current clinical breakpoints suggested [20,21], which define resistance as >1 mg/L of the antibiotics.

### 2.3. Determination of Interactions Between TEO and Antibiotic Compounds

Table 2 describes the results of the interaction between TEO and ampicillin, gentamicin, and penicillin G, demonstrating a reduction in MIC and MBC values in several cases. In detail, a reduction in MICs was observed when ampicillin and TEO were combined against *L. monocytogenes* 120 and 229, where the MICs and MBCs of the antibiotic decreased, respectively, from 8 to 1 and from 2 to 1 µL/mL after 48 h at 37 °C, lowering the value below the breakpoint (≤1 mg/L) suggested by EUCAST [21]. Similarly, the combination of gentamicin with TEO resulted in a decrease in the concentration required to inhibit *L. monocytogenes* 120 and 229, with MIC and MBC reductions from 2 to 1 µL/mL, bringing the value within the breakpoint generally suggested (≤1 mg/L) [20]. Contextually, penicillin G with TEO showed a reduction in MICs and MBCs from 64 to 32 µL/mL and from 4 to 1 µL/mL against *L. monocytogenes* 6 and 229, respectively.

**Figure 1:**
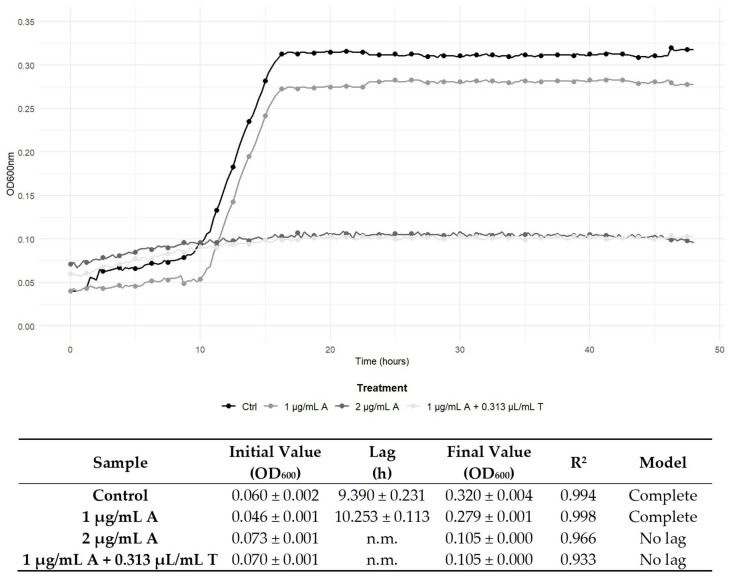
Growth curves of *L. monocytogenes* 120 during 48 h of incubation at 37 °C in control conditions (no antimicrobials), with ampicillin (A) at Minimum Inhibitory Concentrations (MICs, 2 μg/mL) and sub-inhibitory concentrations (MIC/2, 1 μg/mL), and with the combined treatment of ampicillin and TEO (T), both at their combined MIC (1 μg/mL/0.313 μL/mL). Below the graph, the growth parameters obtained by modelling the curves according to the Baranyi and Roberts equation are reported. n.m.: not modellable.

**Figure 2:**
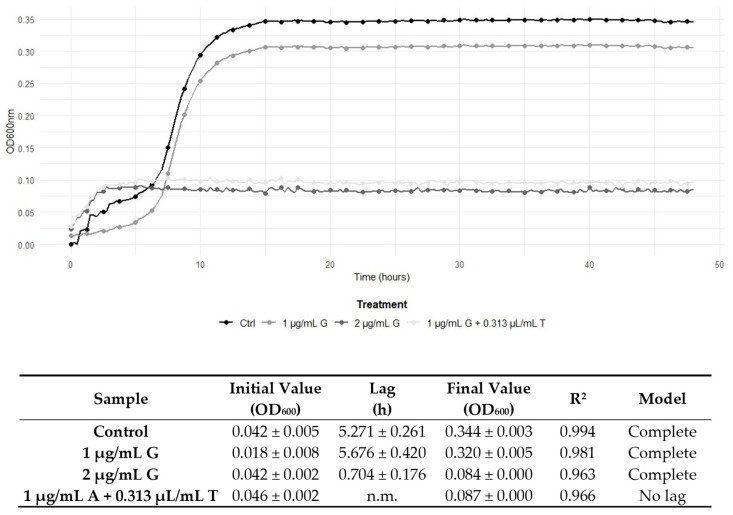
Growth curves of *L. monocytogenes* 120 during 48 h of incubation at 37 °C in control conditions (no antimicrobials), with gentamicin (G) at Minimum Inhibitory Concentrations (MICs, 2 μg/mL) and sub-inhibitory concentrations (MIC/2, 1 μg/mL), and with the combined treatment of ampicillin and TEO (T), both at their combined MIC (1 μg/mL/0.313 μL/mL). Below the graph, the growth parameters obtained by modelling the curves according to the Baranyi and Roberts equation are reported. n.m.: not modellable.

**Figure 3:**
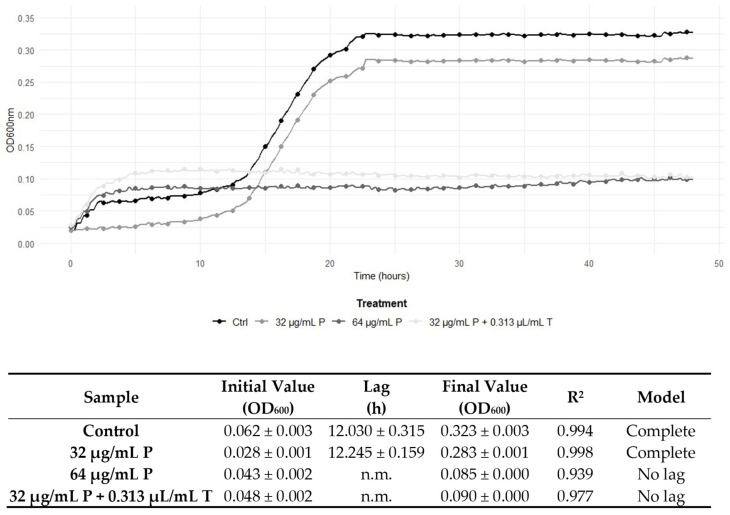
Growth curves of *L. monocytogenes* 6 during 48 h of incubation at 37 °C in control conditions (no antimicrobials), with penicillin G (P) at Minimum Inhibitory Concentrations (MICs, 64 μg/mL) and sub-inhibitory concentrations (MIC/2, 32 μg/mL), and with the combined treatment of ampicillin and TEO (T), both at their combined MIC (32 μg/mL/0.313 μL/mL). Below the graph, the growth parameters obtained by modelling the curves according to the Baranyi and Roberts equation are reported. n.m.: not modellable.

The determination of the FICI enabled the characterisation of the interaction between the antimicrobial compounds. Specifically, Table 3 illustrates the effects of combining TEO with the antibiotic compounds. An additive effect was revealed for the combination of gentamicin and penicillin G with TEO on *L. monocytogenes* 229. The remaining combined treatments exhibited indifferent effects, and it is noteworthy that no antagonistic effects were observed. Interestingly, synergistic effects were observed for the combined treatment with the antibiotics and TEO. In detail, the combination of ampicillin, gentamicin, and penicillin G with TEO determined synergistic effects against *L. monocytogenes* 120, and the same effect was observed for penicillin G and the EO on strain 6. In more recent commentaries about the FIC Index, other authors proposed considering synergism when an FICI value ≤0.50 is obtained [22]. According to this theory, FICI values of 0.63 and 0.75 observed in our study could not indicate real synergism, although the combination of the two antimicrobial compounds still unveils a positive interaction. This prudential definition of synergism is due to the absence of the standardisation of the methods applied, according to Odds et al. [23]. Nevertheless, as suggested by Fratini et al. [24], to reduce the possibility of a mistake, the MIC values to determine FICI values were detected again in the checkerboard method applied and were superimposable with those determined in the previous single analyses. Nonetheless, to better understand the kind of effect directly on the growth kinetics, the development of *L. monocytogenes* strains 120 and 6 was assessed in the presence of ampicillin, gentamicin, and penicillin G and TEO. The addition of 0.313 µL/mL of TEO to *L. monocytogenes* 120 cultures enhanced the antimicrobial effectiveness of both ampicillin and gentamicin, leading to complete growth inhibition at 1 μg/mL, compared to the MIC values of the antibiotics when used alone (2 μg/mL) (Figure 1 and Figure 2). Likewise, 0.313 μL/mL of TEO significantly potentiated the bactericidal activity of penicillin G, as shown by a reduction in the MIC against *L. monocytogenes* strain 6, compared to the MIC value of penicillin G alone (64 μg/mL) (Figure 3). The data were confirmed by the growth parameters reported in Figure 1, Figure 2 and Figure 3. In fact, control samples and cells exposed to MIC/2 had a complete growth curve, with a lag phase length depending on the strain, and a significant increase in the final growth value was observed. On the contrary, cells exposed to the MIC of antibiotics and to the suitable combination of the antibiotics with TEO did not show complete growth curves, while the final growth values obtained depicted a substantial steady condition over time, confirming the growth inhibition. Interestingly, for each reported strain, the parameters obtained for cells exposed to antibiotics at the MIC and to the combination of the antibiotics (below the MIC) with TEO gave superimposable results, suggesting the effectiveness of TEO in reducing the antibiotic concentration necessary to inhibit cell growth. These findings demonstrate that TEO boosts the bactericidal effectiveness of ampicillin, gentamicin, and penicillin G against *L. monocytogenes*.

### 2.4. Determination of the Effect of the Pre-Exposure to a Sub-Inhibitory Concentration of TEO on Antibiotic Compounds’ Effectiveness

Table 4 displays the MIC and MBC values of the antibiotic compounds obtained after a 1 h pre-exposure of the cells to sub-inhibitory concentrations (MIC/2) of TEO. The MICs and MBCs were determined after 48 h of incubation at 37 °C. According to results, pre-exposure to TEO at MIC/2 led to a significant reduction in the MICs and MBCs of antibiotics in most cases. Specifically, *L. monocytogenes* strains 17, 120, and 229 demonstrated increased susceptibility, with lower MICs and MBCs compared to treatment with ampicillin alone (Table 2), reaching the breakpoint established by EUCAST of ≤1 mg/L [21]. The same behaviour was observed with gentamicin and penicillin G against *L. monocytogenes* 120 and 229. *L*. *monocytogenes* strain 6 exhibited increased susceptibility; in this case, a 32-fold reduction in MICs and MBCs was observed after 48 h of incubation at 37 °C, compared to treatment with penicillin G alone (from 64 to 2 μg/mL). An increase in MICs and MBCs following pre-exposure to TEO was observed only in one case. Finally, on the strain 17, gentamicin showed MIC and MBC values of 4 μL/mL after 48 h at 37 °C, compared to 2 μL/mL with gentamicin treatment alone.

### 2.5. Graphical Representation of the Interactions Between TEO and Conventional Antibiotic Treatments

**Figure 4:**
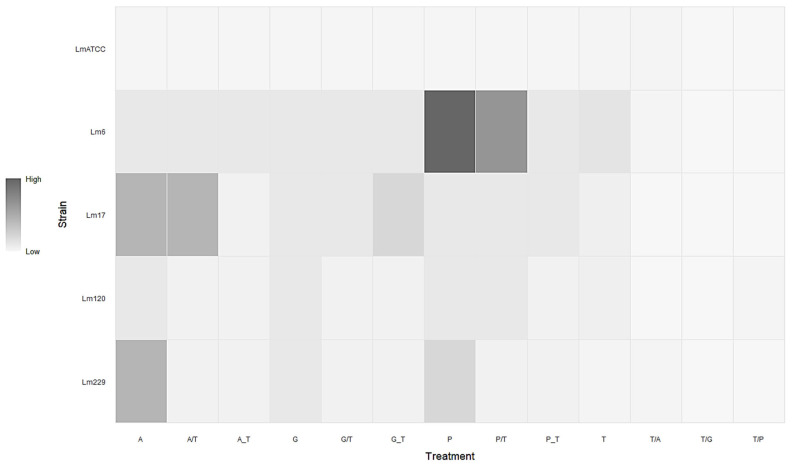
Grey scale-coded matrix of the interactions between TEO and conventional antibiotic treatments in terms of MICs determined after 48 h at 37 °C. Data values are represented by a grey scale gradient, ranging from light grey (lowest MIC values, 0.313 μL/mL) to dark grey (highest MIC values, 64 μg/mL). A: Ampicillin; G: Gentamicin; P: Penicillin G; T: TEO; A/T, G/T, P/T: combination between an antibiotic and TEO; A_T, G_T, P_T: pre-exposure to TEO and a following treatment with an antibiotic; T/A, T/G, T/P: combination between TEO and an antibiotic.

Figure 4 shows the graphical representation of TEO and ampicillin, gentamicin, and penicillin G treatments against the five *L. monocytogenes* strains. The MIC values resulting from treatments with conventional antibiotics alone and in combination (Table 2), and after pre-exposure to TEO for 48 h at 37 °C (Table 4), were plotted into the grey scale-coded matrix. Figure 4 highlights a strain-dependent response to antimicrobial treatments, showing that the combination of TEO with antibiotic compounds and the pre-exposure with EO diminished the MICs of food- and clinical-derived *L. monocytogenes* strains, if compared to antibiotics alone. Interestingly, an evident reduction in MICs was observed for *L. monocytogenes* 6 in the combined treatment and in the pre-exposure to the TEO, with respect to the treatment with penicillin G alone. The combination with TEO did not affect the antimicrobial effectiveness of ampicillin compared to the individual treatment; nevertheless, pre-exposure to EO determined a distinct reduction in ampicillin MIC in *L. monocytogenes* 17. Moreover, *L. monocytogenes* 229 exhibited a reduced ampicillin MIC both in combination and after pre-exposure to TEO. Remarkably, the combination between antibiotics and EO also determined a reduction in the MIC values of TEO in *L. monocytogenes* strains (Figure 4 and Table 2). This behaviour was evident in all the strains under investigation, except for *L. monocytogenes* ATCC 7644 and strain 229 in the presence of ampicillin.

## 3. Discussion

AMR, defined as the adaptation of some microorganisms that acquire the ability to survive or grow in the presence of an antimicrobial agent [25], is a critical global health concern, which jeopardises the effectiveness of infection treatment and prevention [26]. *L. monocytogenes*, as well as other pathogenic bacteria, can exhibit resistance to a wide range of antibiotics [9]; the presence of resistant *L. monocytogenes* strains of food or nosocomial origin has been recently revealed in different geographical areas [27,28]. Therefore, innovative approaches to support and modulate *L. monocytogenes* AMR are of particular importance, given their potential to pose a risk to vulnerable population groups through the transmission of contaminated food products. In this context, the exploitation of natural products, such as EOs, represents a promising alternative for AMR intervention. EOs are lipophilic mixtures composed of diverse bioactive compounds extracted from aromatic plants that confer broad-spectrum antimicrobial activity [29]. The EOs phytocomplex targets multiple bacterial cellular targets, instead of adopting a particular single mode of action, thus reducing the ability of foodborne pathogens to develop resistance [30,31]. Hence, the outcomes of this study suggest the promising potential of TEO to modulate the susceptibility of *L. monocytogenes* to conventional antibiotic treatments. Chemical analysis revealed that carvacrol was the dominant constituent of the TEO (Table 1), corroborating previous studies that have identified carvacrol as a key bioactive compound responsible for antimicrobial activity, mainly determining a modification in the membrane fluidity of *L. monocytogenes* cells and acting as a membrane disrupter, causing the collapse of the proton motive force and the depletion of ATP [11,32]. The associated terpenes, such as *β*-Caryophyllene, *p*-Cymene, and *γ*-Terpinene (Table 1), can contribute to the antimicrobial activity through mechanisms involving the damage to different cellular targets, boosting the antibiotics’ antimicrobial effectiveness [33]. Also, *β*-Caryophyllene increases membrane permeability, causing structural damage and promoting intracellular content leakage [34]. A similar effect on the cell membrane and an influence on the membrane potential has also been described for the monoterpene *p*-Cymene, which is a precursor of carvacrol [35].

In this study, one type strain and four isolates from food and clinical samples were investigated, as the strains could acquire different kinds of resistance depending on the environment they are exposed to. For example, food strains generally require a greater concentration of EOs with respect to clinical strains due to the common use of spices and plant extracts in food formulations [36]. As shown in Table 2, TEO exhibited antimicrobial effectiveness against the *L. monocytogenes* strains tested, particularly notable against *L. monocytogenes* ATCC 7644. Nevertheless, the food- and clinical-derived *L. monocytogenes* strains (6, 17, 120, and 229) displayed higher MIC and MBC values to ampicillin and penicillin G, aligning with the literature that shows increasing resistance trends in *L. monocytogenes* due to horizontal gene transfer and intrinsic resistance mechanisms [4,7]. In fact, ampicillin and penicillin G exhibit preferential inhibition on the same cellular target, which is the bacterial cell wall synthesis [7,9]; instead, gentamicin’s mechanism of action involves the inhibition of bacterial protein synthesis by binding to 30S ribosomes [37]. Interestingly, our study revealed a general reduction in MICs and MBCs when the antibiotics were combined with TEO, suggesting restored susceptibility in *L. monocytogenes* strains (Table 2). The FICI confirmed a positive interaction, particularly in combinations involving ampicillin, gentamicin, and penicillin G against *L. monocytogenes* strains 120 and 6 (Figure 1, Figure 2 and Figure 3). Notably, no antagonistic interactions were observed across any treatment, thereby reinforcing the EO’s potential compatibility with conventional antibiotics. The restoration of antibiotic susceptibility and the synergy between the antibiotic compounds and EO in *L. monocytogenes* may be linked to the main bioactive components of TEO. Carvacrol, when taken with first-line antibiotics, has demonstrated significant synergistic effects against multidrug-resistant bacteria due to damage on the cell membrane inducing the abnormally elongated shapes with ruptured or broken cell walls [38]. The combined effect of antibiotics disrupting cell wall synthesis and of TEO affecting the integrity and functionality of the cytoplasmic membrane could therefore produce a critical effect on cell integrity and viability. It has been suggested that synergistic interactions encompass the progressive inhibition of a shared metabolic pathway and the suppression of protective enzymes, thus augmenting the absorption of cell wall-active drugs, such as ampicillin and penicillin G [39].

While other authors have already combined EOs and antibiotics, in this study, *L. monocytogenes* cells were temporarily exposed to sublethal concentrations of TEO to investigate a potential effect in increasing the susceptibility to the three antibiotics. Indeed, the pre-exposure to sub-inhibitory concentrations of TEO further enhanced antibiotic activity by reducing MIC and MBC values (Table 4). For instance, the MIC of penicillin G against *L. monocytogenes* 6 decreased by 32-fold following TEO pretreatment, inducing an evident reversion to susceptibility. This result suggests that EO preconditioning may modify bacterial stress response pathways or membrane properties, thereby increasing permeability and therefore cellular uptake or reducing efflux pump activity, as hypothesised for other EOs chemotyped to carvacrol or thymol, or for carvacrol alone in similar studies [12,17]. In particular, it has been demonstrated that the depolarization of the membrane induced by carvacrol, the main constituent of TEO, may promote the aminoglycosides’ entrance within the cytoplasm, thus enhancing their effects [17]. Moreover, carvacrol seemed to act as an efflux pump inhibitor in *S. aureus* and hence hampered the cells’ reactions to antibiotics, making them more susceptible [17].

Only one exception was observed, where *L. monocytogenes* 17 exhibited increased MIC and MBC values following TEO pre-exposure in gentamicin treatment (Table 4), supporting a modulatory role of the EOs on the antibiotic compounds’ activity, underscoring the strain-specific responses and highlighting the complexity of EO–antibiotic interactions, which require further molecular-level investigation. It is noteworthy that the effect of the pre-exposure to the EO in reducing antibiotics’ MIC and MBC values was particularly clear on clinical strains, encouraging the exploitation of EOs in clinical practice as well, where AMR is a diffused and alarming phenomenon. Finally, when TEO was employed in combination with or as a pre-exposure to conventional antibiotics, a general reduction in its MIC values was observed (Figure 4 and Table 2). These findings are particularly valuable considering the different intracellular targets of the two kinds of treatments. From the perspective of mitigating AMR in *L. monocytogenes*, this observation may demonstrate significant utility for generally compromising bacterial cellular integrity while concurrently enabling the administration of reduced antimicrobial agent concentrations, thereby potentially attenuating associated adverse effects.

Combining EOs and antibiotics has been shown to enhance antimicrobial effectiveness by lowering the required effective concentrations. Furthermore, other authors observed that sublethal concentrations of carvacrol can prevent the emergence of resistance when used in association with aminoglycosides [17]. Nevertheless, potential limitations such as cytotoxicity must be carefully considered. While several studies have reported reduced cytotoxic effects associated with EOs, attributed to the low concentrations needed for antimicrobial activity [15,18,40], conversely, the combined use of antibiotics and EOs may exert antimicrobial effects at cytotoxic concentrations [40]. To date, the interactions between the different treatments and their associated cytotoxicity remain insufficiently explored, and this highlights the need for further investigation.

The results observed align with evidence supporting the use of EOs to modulate AMR in *L. monocytogenes* due to the multifaceted mechanism of action, suggesting EOs as a candidate for integration into antimicrobial strategies targeting multidrug-resistant pathogens. Future research should focus on elucidating the molecular mechanisms underlying the EO-induced susceptibility restoration and the interactions with conventional antibiotics, exploring gene expression changes, efflux pump inhibition, and membrane permeability alterations by using cutting-edge-omics approaches. Nonetheless, strategies to combine the two different antimicrobials in clinical practice should be further developed.

## 4. Materials and Methods

### 4.1. Antimicrobic Compounds

The commercial, food-grade TEO was kindly supplied by Exentiae s.r.l. (Catania, Italy). The EO emulsions were formulated by diluting to 80 µL/mL in 10 mM of Phosphate-Buffer Saline (PBS), with a pH of 7.4, and including 10 µL/mL of Tween 80 (Sigma-Aldrich, Milan, Italy). Lyophilised ampicillin, gentamicin, and penicillin G were acquired by Sigma-Aldrich (Milan, Italy), and the solutions were prepared by diluting the antibiotics in distilled water to achieve a concentration of 512 µg/mL.

### 4.2. Gas Chromatography-Based Analysis

To chemically characterise TEO, the analyses were carried out on a Clarus 500 model Perkin Elmer (Waltham, MA, USA) gas chromatograph coupled with a mass spectrometer equipped with an FID (flame ionisation detector). The chosen capillary column was a Varian Factor Four VF-5. The GC oven programmed temperature was set initially at 60 °C and then increased to 220 °C at 6°/min and finally held for 20 min. Helium was used as a carrier gas at a constant rate of 1 mL/min. MS detection was performed with electron ionisation (EI) at 70 eV operating in the full-scan acquisition mode in the m/z range of 40–550 amu. The identification of compounds was performed by the comparison of the MS-fragmentation pattern of the analytes with those of pure components stored in the Wiley 2.2 and Nist 11 mass spectra libraries database. Further, the Linear Retention Indices (LRIs) were calculated using a series of alkane standards (C_8_–C_25_ *n*-alkanes). The obtained LRIs were compared with available retention data reported in the literature. The relative amounts of the components were expressed as the percent peak area relative to the total peak area without the use of an internal standard and without any factor correction. All analyses were carried out in triplicate.

### 4.3. Bacterial Strains and Culture Conditions

Five *L. monocytogenes* strains from different origins were studied (Table 5). Type strain ATCC 7644, and strains 6 and 17 belong to the collection of the Department of Bioscience and Technology for Food, Agriculture, and Environment at Teramo University, Italy. Strains 120 and 229 were kindly provided by Santo Spirito Hospital in Pescara, Italy. The fresh cultures grown on Brain Heart Infusion (BHI) agar plates (Liofilchem, Roseto degli Abruzzi, Italy) were inoculated into BHI broth (Liofilchem) and incubated at 37 °C for 18 h to achieve an early stationary phase fresh culture. The standardised inocula were acquired through measurement with a Lambda Bio 20 spectrophotometer (PerkinElmer, Waltham, MA, USA) and subsequently diluted to 10^8^ CFU/mL.

### 4.4. Determination of Minimum Inhibitory Concentrations and Minimum Bactericidal Concentrations

The MICs of the EO and antibiotic compounds were evaluated using the microdilution method [41] in a 96-well microtiter plate (Corning Incorporated, Kennebunk, ME, USA) at 37 °C after 24 and 48 h. The Omnilog incubator/reader (Biolog Inc., Hayward, CA, USA) tracked the bacterial growth by scanning the plates every 15 min to gather kinetic data, which were then converted into optical density (OD_600 nm_) and further analysed. MIC values were defined as the minimal concentration of antimicrobial agents at which no growth was observed. Thereafter, the MBCs were ascertained by streaking cells onto Mueller–Hinton agar plates after 24 and 48 h at 37 °C.

The analysis was conducted in three biological replicates and expressed as the means of the repetitions.

### 4.5. Determination of Synergy Between TEO and Antibiotic Compounds

The interaction resulting from the combination of TEO with ampicillin, gentamicin, and penicillin G was determined using the checkerboard assay [24]. The FICI was computed using Equation (1), facilitating the classification of the interaction between the two antimicrobial compounds as synergistic (FICI ≤ 1.0), additive (FICI = 1), indifferent (1.0 < FICI ≤ 2.0), or antagonistic (FICI > 2.0).

FICI = *FIC_A_ + **FIC_B_ (1)

*FIC_A_ = MIC_A_ in combination/MIC_A_ alone,

**FIC_B_ = MIC_B_ in combination/MIC_B_ alone.

### 4.6. Determination of the Effect of Sub-Lethal Concentrations of TEO Pre-Exposure on Antibiotic Compounds Treatments

The effect of pre-exposure to TEO on the antibiotic susceptibility of *L. monocytogenes* strains under study was assessed by determining the MICs and MBCs. Suspensions of *L. monocytogenes* strains, containing approximately 5 × 10^8^ CFU/mL in 10 mM of PBS buffer solution at a pH of 7.4, were prepared in the presence of TEO at sub-inhibitory concentrations, determined as MIC/2 and therefore different for each strain. The pretreated *L. monocytogenes* suspensions were then vortexed and incubated at 37° C for 1 h. Following incubation, the cells were washed three times with 10 mM of PBS (pH 7.4) to remove any residue of TEO. Then, the *L. monocytogenes* suspensions were treated with ampicillin, gentamicin, and penicillin G to determine the MICs and MBCs by the microdilution method [41], as reported in Section 4.3.

### 4.7. Data Analysis

All statistical analyses and visualisations were performed by R statistical software (version 4.3.1; R Core Team, 2023) [42] and the RStudio IDE (version 2024.12.1; RStudio Team, 2025) [43]. The following R packages were used for data manipulation and visualisation: readxl (version 1.4.4) [44]; dplyr (version 1.1.2) [45]; ggplot2 (version 3.5.1) [46]; and tidyr (Version 1.3.0) [47].

Selected growth data were obtained by the Omnilog reader, and the means of the three replicates were calculated. The data were further processed by fitting the growth curves by means of Baranyi and Roberts equations [47] with DMFit software (available at www.combase.cc; last accessed on 18 May 2025).

## 5. Conclusions

The results highlight the potential of TEO as a promising adjuvant in antimicrobial strategies targeting *L. monocytogenes* and underline the importance of exploring natural compounds in the fight against resistant microorganisms. The demonstrated synergistic interactions with conventional antibiotics reduce the effective doses required, thereby potentially minimising their adverse effects and the risk of multi-drug resistance, which are associated with their high doses when used alone. Synergistic and additive effects were observed when the TEO was combined with ampicillin, gentamicin, and penicillin G, resulting in reduced MICs and MBCs and in the restoration of susceptibility according to clinical breakpoints. These findings support the concept of dual-action strategies for resistance modulation and confirm that EOs can restore the susceptibility of resistant bacteria to antibiotics. Therefore, EOs can be considered a promising complementary approach to boost the effectiveness of conventional antimicrobial compounds. A more profound understanding of the interactions among the bioactive components could address synergistic combinations in medicinal and food preservation applications. Further research is needed to explore the molecular mechanisms behind the synergistic effects and to assess the potential for applying the combined treatments in both food safety measures and clinical environments.

## Figures and Tables

**Table 1:** Chemical composition (percentage mean value ± SD) of TEO.

N°	Components ^1^	LRI ^2^	LRI ^3^	%
1	*α*-pinene	925	928	1.2 ± 0.06
2	camphene	958	961	0.4 ± 0.02
3	*β*-thujene	971	968	1.8 ± 0.07
4	sabinene	975	972	0.7 ± 0.03
5	*β*-pinene	982	980	0.1 ± 0.01
6	*β*-myrcene	993	992	2.4 ± 0.04
7	*α*-phellandrene	995	998	0.6 ± 0.03
8	3-carene	1012	1014	0.2 ± 0.02
9	*p*-cymene	1020	1016	7.7 ± 0.10
10	*α*-terpinene	1015	1019	2.6 ± 0.07
11	limonene	1025	1022	0.6 ± 0.03
12	1,8-cineole	1036	1033	0.1 ± 0.01
13	*cis*-*β*-ocimene	1041	1037	0.1 ± 0.02
14	*γ*-terpinene	1071	1065	7.6 ± 0.11
15	*p*-cymenene	1086	1083	0.1 ± 0.01
16	terpinolene	1088	1085	0.4 ± 0.02
17	linalool	1096	1095	2.4 ± 0.05
18	terpinen-4-ol	1158	1161	2.3 ± 0.08
19	(*E*)-dihydrocarvone	1210	1207	0.3 ± 0.02
20	neral	1218	1220	0.1 ± 0.01
21	carvacrol	1282	1278	55.4 ± 6.02
22	*β*-caryophyllene	1422	1424	8.6 ± 0.15
23	aromadendrene	1465	1460	0.1 ± 0.01
24	humulene	1471	1473	0.3 ± 0.02
25	*β*-bisabolene	1498	1495	1.5 ± 0.08
26	*δ*-cadinene	1517	1515	0.1 ± 0.02
27	*trans*-*α*-bisabolene	1541	1536	1.2 ± 0.05
28	caryophyllene oxide	1579	1580	1.0 ± 0.06
29	isoaromadendrene epoxide	1591	1594	0.1 ± 0.02
	**SUM**			100.0
	**Monoterpenes**			87.1
	**Sesquiterpenes**			12.9
	**Others**			

^1^ The components are reported according to their elution order on an apolar column; ^2^ Linear Retention Indices are measured on an apolar column; ^3^ Linear Retention indices are from the literature.

**Table 2:** Minimum Inhibitory and Bactericidal Concentrations of ampicillin, gentamicin, penicillin G compounds, and TEO and their combination against the *L. monocytogenes* strains within this study after 48 h at 37 °C.

	A		G		P		T		A/T		G/T		P/T	
	MIC	MBC	MIC	MBC	MIC	MBC	MIC	MBC	MIC	MBC	MIC	MBC	MIC	MBC
	48 h	48 h	48 h	48 h	48 h	48 h	48 h	48 h	48 h	48 h	48 h	48 h	48 h	48 h
*L. m.* ATCC 7644	0.5	0.5	0.5	0.5	0.5	0.5	0.63	0.63	0.5–0.63	0.5–0.63	0.5–0.31	0.5–0.31	0.5–0.31	0.5–0.31
*L. m.* 6	2	2	2	2	64	64	2.5	2.5	2−0.63	2−0.63	2−0.31	2−0.31	32−0.31	32−0.31
*L. m.* 17	8	8	2	2	2	2	1.25	1.25	8−0.31	8−0.31	2−0.31	2−0.31	2−0.31	2−0.31
*L. m.* 120	2	2	2	2	2	2	1.25	1.25	1−0.31	1−0.31	1−0.31	1−0.31	2−0.63	2−0.63
*L. m.* 229	8	8	2	2	4	4	0.63	0.63	1−0.63	1−0.63	1−0.31	1−0.31	1−0.31	1−0.31

Minimum Inhibitory and Bactericidal Concentrations are reported in μL/mL for TEO and in μg/mL for antibiotic compounds. The standard deviation is not included, as it was zero for all replicates. MIC: Minimum Inhibitory Concentration; MBC: Minimum Bactericidal Concentration; A: Ampicillin; G: Gentamicin P: Penicillin G; T: TEO.

**Table 3:** Fractional Inhibitory Concentration Index and effects resulting from the combination of ampicillin, gentamicin, and penicillin G compounds with TEO against the *L. monocytogenes* strains within this study after 48 h at 37 °C.

	A/T	G/T	P/T	A/T	G/T	P/T
	FICI	FICI	FICI	Effect	Effect	Effect
	48 h	48 h	48 h	48 h	48 h	48 h
*L. monocytogenes* ATCC 7644	2	1.5	1.5	IndE	IndE	IndE
*L. monocytogenes* 6	1.25	1.13	0.63	IndE	IndE	SynE
*L. monocytogenes* 17	1.25	1.25	1.25	IndE	IndE	IndE
*L. monocytogenes* 120	0.75	0.75	1.5	SynE	SynE	IndE
*L. monocytogenes* 229	1.12	1	1	IndE	AddE	AddE

FICI: Fractional Inhibitory Concentration Index; A: Ampicillin; G: Gentamicin; P: Penicillin G; T: *Thymbra capitata* L. (Cav.) EO; SynE: Synergism; AddE: Commutative; IndE: Indifference.

**Table 4:** Minimum Inhibitory and Bactericidal Concentrations of ampicillin, gentamicin, and penicillin G compounds after 1 h of pre-exposure to sub-lethal concentrations of TEO of the *L. monocytogenes* strains, observed after 48 h at 37 °C.

	T	A		G		P	
	MIC/2	MIC	MBC	MIC	MBC	MIC	MBC
	48 h	48 h	48 h	48 h	48 h	48 h	48 h
*L. monocytogenes* ATCC 7644	0.31	0.5	0.5	0.5	0.5	0.5	0.5
*L. monocytogenes* 6	1.25	2	2	2	2	2	2
*L. monocytogenes* 17	0.63	1	1	4	4	2	2
*L. monocytogenes* 120	0.63	1	1	1	1	1	1
*L. monocytogenes* 229	0.31	1	1	1	1	1	1

Sub-inhibitory concentrations (MICs/2) are reported in μL/mL. Minimum Inhibitory and Bactericidal Concentrations are reported in μg/mL. The standard deviation is not included, as it was zero for all replicates. MIC/2 refers to the sub-lethal concentration of TEO used for pre-exposure; MICs: Minimum Inhibitory Concentrations; MBCs: Minimum Bactericidal Concentrations; T: TEO; A: Ampicillin; G: Gentamicin; P: Penicillin G.

**Table 5:** *Listeria monocytogenes* strains under study.

Species	Strain Code	Origin
*L. monocytogenes*	ATCC 7644	Type strain
*L. monocytogenes*	6	Pork ribs
*L. monocytogenes*	17	Fermented pork meat-based product
*L. monocytogenes*	120	Clinical isolation from humans
*L. monocytogenes*	229	Clinical isolation from humans

## Data Availability

The original contributions presented in this study are included in the article. Further inquiries can be directed to the corresponding author.
